# Resolving length-scale-dependent transient disorder through an ultrafast phase transition

**DOI:** 10.1038/s41563-024-01927-8

**Published:** 2024-06-13

**Authors:** Jack Griffiths, Ana F. Suzana, Longlong Wu, Samuel D. Marks, Vincent Esposito, Sébastien Boutet, Paul G. Evans, J. F. Mitchell, Mark P. M. Dean, David A. Keen, Ian Robinson, Simon J. L. Billinge, Emil S. Bozin

**Affiliations:** 1 Condensed Matter Physics and Materials Science Division, Brookhaven National Laboratory, Upton, NY USA; 2 Department of Materials Science and Engineering, University of Wisconsin, Madison, WI USA; 3 SLAC National Accelerator Laboratory, Menlo Park, CA USA; 4 Materials Science Division, Argonne National Laboratory, Lemont, IL USA; 5 ISIS Neutron and Muon Source, STFC Rutherford Appleton Laboratory, Didcot, UK; 6 London Centre for Nanotechnology, University College London, London, UK; 7 Department of Applied Physics and Applied Mathematics, Columbia University, New York, NY USA

**Keywords:** Phase transitions and critical phenomena, Structural properties, Characterization and analytical techniques

## Abstract

Material functionality can be strongly determined by structure extending only over nanoscale distances. The pair distribution function presents an opportunity for structural studies beyond idealized crystal models and to investigate structure over varying length scales. Applying this method with ultrafast time resolution has the potential to similarly disrupt the study of structural dynamics and phase transitions. Here we demonstrate such a measurement of CuIr_2_S_4_ optically pumped from its low-temperature Ir-dimerized phase. Dimers are optically suppressed without spatial correlation, generating a structure whose level of disorder strongly depends on the length scale. The redevelopment of structural ordering over tens of picoseconds is directly tracked over both space and time as a transient state is approached. This measurement demonstrates the crucial role of local structure and disorder in non-equilibrium processes as well as the feasibility of accessing this information with state-of-the-art XFEL facilities.

## Abstract (web summary)

Tracking local material structure at picosecond timescales is realized using XFEL facilities. This pair distribution function measurement tracks the redevelopment of structural order through a non-equilibrium transition.

## Main

The development of materials with specialized and highly efficient properties increasingly relies on complex local structures that stray from the ideal of a perfect crystal^1–3^. In particular, advancements in electronics technology drive a need for materials that switch between distinct states: either electrical (for example, memristors^4^, ferroelectrics^5^), magnetic (for example, ferromagnets^6^, anti-ferromagnets^7^) or structural (for example, charge density wave states^8^). A key example is the metal–insulator transition^9,10^. It is well established that local structure plays a central role in many equilibrium phase transitions driven by the competition of energy and entropy^11–13^. Some non-equilibrium phase transitions can be triggered on demand using ultrafast laser pulses. Local structure has also been implicated in these transitions^14,15^, but this is less understood due to a lack of appropriate means to quantify length-scale-dependent local disorder in these ultrafast transient states.

In studies at equilibrium, the pair distribution function (PDF) plays an integral role in characterizing locally broken structural symmetry and structural disorder^16,17^. This function of interatomic distances in the scattering material, generated through a Fourier transform of an appropriately normalized scattering pattern, is a quantitative and easily interpretable probe for atomic structure on all length scales from local to bulk. X-ray free electron laser (XFEL) facilities offer a key opportunity to apply the PDF technique to picosecond structural dynamics, such as phase transitions, using high-brilliance 100 fs X-ray pulses. This would represent a 10^9^ times increase in the temporal resolving power compared with a typical synchrotron experiment that does not severely compromise brilliance with slicing or fast shutter methods^18^. Compared with electrons, which can also be generated in ultrashort pulses, X-rays scatter kinematically and can generate quantitatively reliable PDFs.

Here we demonstrate the feasibility of XFEL ultrafast PDF (uf-PDF) to track an optically pumped phase transition in CuIr_2_S_4_ (CIS). This measurement shows that although the local atomic structure transitions in less than a picosecond, the average structure on length scales longer than a unit cell continues to strongly evolve for tens of picoseconds as long-range order between local regions is established. Although the pumping process produces a transition between two ordered phases, this measurement tracks the pivotal role of disorder through the transition itself.

**Fig. 1:**
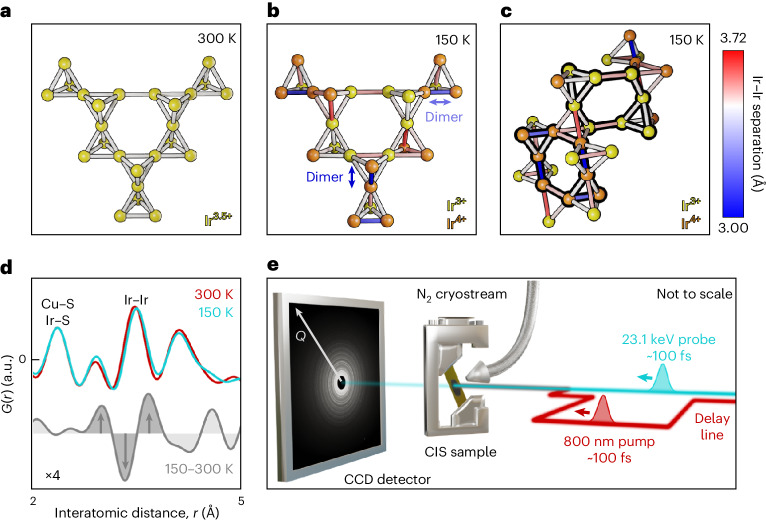
CIS Ir dimerization. **a**, Portion of the undistorted pyrochlore Ir substructure in the 300 K CIS structure described in space group symmetry ${{Fd}}\bar{3}{{m}}$. Structure refined from experimental data. **b**, Portion of the CIS Ir substructure at 150 K as described in the triclinic space group ${{P}}\bar{1}$. Charge/orbital ordering and spin dimerization separates Ir–Ir bonds into substantially shortened (blue) and lengthened (red) or largely unaltered (grey) groups. Dimerized bonds run along two distinct [110]-type cubic directions (examples marked by arrows). Structure refined from experimental data. **c**, Different portions and views of the same 150 K CIS Ir substructure, emphasizing topologically equivalent bicapped hexamers containing either Ir^3+^ or Ir^4+^ ions (bold outlines). **d**, Ir dimerization creates an M-shaped differential PDF signature at the length scale of an Ir–Ir bond (arrows). PDFs measured at the LCLS XFEL in equilibrium. Differential magnified four times for clarity. **e**, Experimental schematic of the XFEL pump–probe uf-PDF measurements of low-temperature CIS. Pump delay line is able to adjust the relative pump–probe arrival time at the sample from –20 ps (maximum pump delay) to 100 ps (minimum pump delay). CCD, charge-coupled device.

CIS is a spinel material exhibiting a metal–insulator phase transition (generally described as Peierls-like) on cooling through 226 K (ref. ^19^). Above this temperature, it consists of a cubic ${{Fd}}\bar{3}{{m}}$ unit cell with Ir atoms, the dominant X-ray scatterers, forming a pyrochlore substructure of regular tetrahedra with an edge length of ~3.5 Å (Fig. 1a). This generates a strong PDF peak at this interatomic distance. Below 226 K, the Ir undergoes charge and orbital ordering as their effective 3.5+ charge disproportionates to a nominally 3+/4+ state. Simultaneously, spin dimerization (Fig. 1b,c) shortens (dimers) and lengthens some Ir–Ir distances with a separation in length of ~0.7 Å. The resulting M-shaped signature in the difference of PDF profiles across the transition is large enough to be well resolved with the limited resolution of an XFEL measurement (Fig. 1d). The dramatic loss in symmetry through this transition is reflected by a new triclinic ${{P}}\bar{1}$ unit cell^19^. The Ir dimerization can be described by chains running along two distinct [110]-type cubic directions^20^ (Fig. 1b, arrows) or, alternatively, by two topologically identical eight-atom bicapped hexamers (hereafter referred to as Ir octamers)^19^. These consist either of Ir^4+^ (containing dimerized bonds) or Ir^3+^ (containing non-dimerized bonds) ions and together tile three-dimensional space (Fig. 1c, bold outlines). In 2019, high-resolution synchrotron PDF was used to investigate how the local structure of CIS harbingers the bulk phase transition^21^. Although the metallic phase is cubic on average, each Ir–Ir bond dynamically fluctuates by <0.1 Å due to an orbital degeneracy lifting (ODL) precursor effect that reduces the local symmetry to tetragonal *I*4_1_/*amd* (a subgroup of ${{Fd}}\bar{3}{{m}}$). These ODL dimers, which are correlated over increasing distances as the phase transition is approached, cast doubt on the Peierls mechanism of the metal–insulator transition and suggest a greater complexity that is unlikely to be understood from equilibrium structural measurements.

Ultrafast reflectivity studies^22,23^ have shown that dimerized CIS responds to optical pumping with reflectivity decreasing and recovering over sub-picosecond and tens of picosecond timescales, respectively. Using multipulse techniques, it has been argued that the (so far unidentified) pumped phase represents a distinct transient structure and not a return to the high-temperature ${{Fd}}\bar{3}{{m}}$ state^22^. The pumped phase is weakly conducting, suggesting the removal of strong dimerization, and could therefore be speculatively related to an ordered variant of the ODL state. As the removal of strong dimerization in CIS would generate a large-enough PDF signal to be clearly resolvable in an XFEL measurement (Fig. 1d), this pumped transition is ideal for appraising the uf-PDF technique while simultaneously examining how CIS transitions between the insulating and conducting states. Note that dimerized CIS is also known to be sensitive to continuous irradiation by ultraviolet or X-ray photons over hundreds of milliseconds^24–27^. In this markedly distinct regime of photon energies and peak fluences, the X-ray Bragg scattering signature of dimers is removed. However, PDF has shown that only the long-range dimer order is destroyed, whereas the dimers themselves persist locally^25^. In the work demonstrating this^25^, the number of local dimers that could have been possibly destroyed and not resolved is, extremely conservatively, 10%.

To investigate this optically driven transition, a layer of powdered CIS was pumped at 150 K using an 800 nm laser pulse. The pumped sample was stroboscopically probed with X-ray pulses using a transmission scattering geometry at the Macromolecular Femtosecond Crystallography (MFX) beamline of the Linac Coherent Light Source (LCLS) XFEL facility (Fig. 1e and Methods). A scattering momentum transfer range from 1.6 to 12.6 Å^−1^ was achieved using 23.1 keV photons. This can be compared with other current XFEL scattering and PDF (without time resolution) measurements, achieving only maximum momentum transfers of 5–8 Å^−1^ that severely limit the real-space resolution and degrade the information needed for a quantitative analysis^14,28,29^. A reference (unpumped) measurement of the sample was also taken at the same temperature at the Advanced Photon Source synchrotron with a larger maximum momentum transfer of 23 Å^−1^.

## Results

**Fig. 2:**
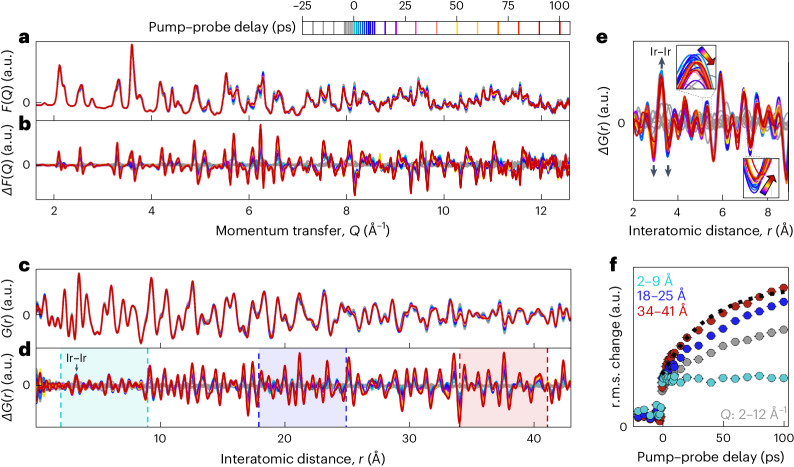
Structural response to laser pump. **a**, Reduced structure factor *F*(*Q*), a linear function of the diffraction pattern that magnifies high-*Q* features, with varying pump–probe delays. All the negative delay signals (unpumped measurements) are coloured in grey. **b**, Δ*F*(*Q*) subtracting the average of {–20, –15, –10} ps to emphasize differences due to laser pump. The *y* axis is magnified 3.4 times relative to *F*(*Q*). **c**, PDF *G*(*r*) with varying pump–probe delays. **d**, Δ*G*(*r*) subtracting the average of {–20, –15, –10} ps to emphasize differences due to laser pump. The *y* axis is magnified 3.4 times relative to *G*(*r*). **e**, Δ*G*(*r*) over the sub-nanometre length scales showing a sub-picosecond abrupt change. The signature of Ir–Ir dimer suppression, the inverse of ‘M’ (Fig. 1d), is marked with black arrows. The insets show that smaller changes to the PDF continue with increasing delay. **f**, The r.m.s. value of Δ*G*(*r*) calculated over equally sized ranges of interatomic distance, showing the same initial prompt response at 0 ps but a difference in further structural evolution at local and non-local length scales. Δ*G*(*r*) r.m.s. value over the 34–41 Å range follows an exponential behaviour with (38 ± 6) ps characteristic time (black dashed line). The r.m.s. value of Δ*F*(*Q*) is also shown (grey), representing all the accessible length scales. Δ*F*(*Q*) normalized to match negative delay values for Δ*G*(*r*) (representing the noise level). The error bars indicate uncertainty only due to photon shot noise.

Initial observations regarding the structural response to the pump laser can be made in reciprocal space. The reduced structure factors *F*(*Q*) (where *Q* is the scattering momentum transfer) and the difference curves Δ*F*(*Q*) (where an averaged unpumped reference is subtracted to amplify more subtle changes) are shown as a function of the pump–probe delay (Fig. 2a,b). There is a clear abrupt change in the pattern on crossing 0 ps pump–probe delay, indicating a structural response. These changes then evolve smoothly in time over the measured *Q* range and do not begin to reverse in the 100 ps measurement time, indicating a lifetime of at least this long for the pumped structural phase. These observations suggest that the pumping process can be described in terms of three structures: the dimerized structure before pumping (which is well characterized), the prompt structure immediately on pumping (characterized in this work) and the transient structure that the material relaxes to over tens of picoseconds (also characterized here). Importantly, no substantial response—including heating-induced lattice expansion—was observed when pumping the high-temperature phase (Supplementary Figs. 1 and 2).

PDFs *G*(*r*) (where *r* is the interatomic distance) are generated from sine Fourier transforms of the reduced structure factor (Fig. 2c,d), where the finite range of measured *Q* applies a well-understood convolution that broadens peaks and introduces termination ripples. Despite these termination artefacts, analysing the data in real space has several key advantages. First, subtle and/or broadband changes to diffuse scattering are converted to changes in the positions and shapes of peaks that are easier to identify, interpret and model. Second, focusing on different regions of a PDF reveals how the average atomic structure varies over different length scales. This provides information on structural ordering. Here the difference curves Δ*G*(*r*) display the W signature that some strong Ir–Ir dimers are removed by the laser pump (Fig. 2e (arrows), opposite to the M feature (Fig. 1d (arrows))). The central positive peak of this ‘W’ along with the two adjacent negative valleys represents a reduction in the spread of Ir–Ir distances as they shift towards a central value. As this signature is fully formed at 1 ps pump–probe delay, this strong-dimer suppression must occur on femtosecond timescales and is not probed temporally here. This is consistent with optical dimer removal in VO_2_, for example, which occurs over 100 fs (ref. ^14^). The shape of the sub-nanometre response bears a strong qualitative agreement to a simple calculation of multiple unit cells in which either a single or all of the Ir–Ir dimers are lengthened by 0.4 Å (Supplementary Note 1).

The >1 ps time evolution of the pumped PDFs strongly depends on length scale *r*. Following the laser pump, the features in the difference curve Δ*G*(*r*) are initially similar in scale at all *r* (Fig. 2d). With continuing delay, these scales evolve differently above and below ~9 Å—the length scale of one unit cell/octamer (Supplementary Fig. 3). Although the PDF below 9 Å, and therefore the distribution of nearest-neighbour atomic distances, could be tentatively argued to continue to subtly evolve with delay just above the noise level (Fig. 2e, insets), any changes are dwarfed by the initial abrupt response. This is clear from the constant root mean square (r.m.s.) of Δ*G*(*r*) from 2 to 9 Å, reducing the information in Δ*G*(*r*) to a simple magnitude of PDF change (Fig. 2f). In contrast, this same metric at longer length scales (18–25 Å and 34–41 Å) features a similar initial step change followed by strong growth incomplete within 100 ps. This implies that the pumped prompt and transient phases are the same on local length scales but distinct over length scales averaging over multiple unit cells. Both differ from the starting dimerized phase at all length scales. The longer timescale increase in the r.m.s. metric over 34–41 Å follows an exponential time constant of (38 ± 6) ps.

A picture emerges from these real-space observations. The structural changes instigated by the optical removal of Ir dimers (a stochastic process) are initially uncorrelated between local regions of the sample. That is, in the prompt phase, the spatial arrangement of bond lengths differs from one local region to the next. Internal strain imposed by this disorder probably drives the redevelopment of a non-equilibrium long-range order over time. This alters the PDF over longer length scales as the transient phase is approached while leaving the local PDF over sub-unit-cell length scales largely unchanged. A minor evolution of the local PDF with delay could be permitted as the external strain on each unit cell reduces with decreasing disorder. Note that the observation of the removal of strong dimers precludes the pumped state from being the same as that reached under continuous ultraviolet or X-ray irradiation in which local dimerization is preserved^25^.

The average W signature centred at 3.5 Å in Δ*G*(*r*) can be compared with the same signature generated by the equilibrium (thermally driven) transition in which all the strong dimers are removed. Although qualitatively very similar, the thermally driven signature must be scaled by ~0.29 times to match the pumped signature intensity. This indicates that ~29% of probed dimers in the pumped sample are suppressed. As the pump laser is expected to penetrate 40 nm into each ~0.7 μm powder grain^22^, we would expect only a maximum of ~4% of probed dimers to be suppressed depending on the fraction of dimer suppression within the pumped volume. This suggests that either (1) the characterization of the powder was inaccurate and the average grain size is smaller than that determined by confocal microscopy (Supplementary Fig. 4) or (2) the transition occurs within a greater depth with energy carried by, for example, non-thermal photoexcited electrons^30,31^. Although the pumped phase fraction cannot be unambiguously determined, there is a lower bound of ~29% on the proportion of dimers suppressed within the pumped volume.

**Fig. 3:**
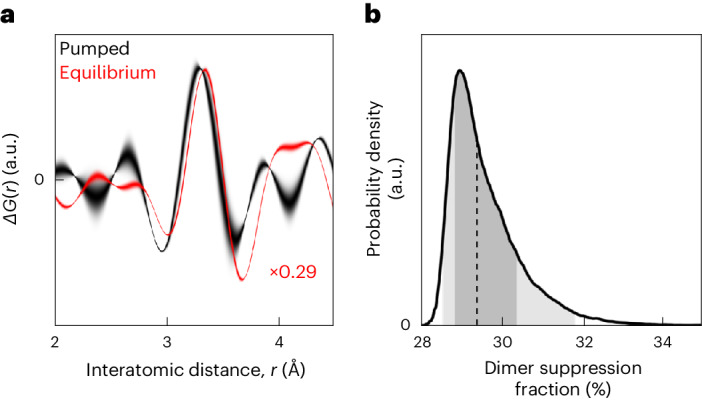
Dimer suppression signature. **a**, Experimental Δ*G*(*r*) for both pumped (black) and thermally driven equilibrium (red) transitions, showing the dimerized PDF subtracted from the un-dimerized PDF, over the low-*r* range containing the W signature centred at 3.5 Å of strong-dimer removal. The pumped data averages over all the positive pump–probe delay times, with the line thickness indicating uncertainty (Supplementary Note 2). The equilibrium data are scaled by 0.29 times; therefore, the signatures approximately match in scale. **b**, Uncertainty in PDF normalization is propagated to a probability density function representing scaling between the pumped and equilibrium dimer suppression signatures (Supplementary Note 2). The dashed line indicates the median probable value of 29.4%. The shaded areas indicate the central 68% and 95% probability intervals.

The pumped signature can also be compared with a simple ‘small-box’ model, a numerically generated PDF of a perfect crystal (with experimental effects such as termination artefacts reproduced) that is typically compared with an experimental PDF over a certain interatomic distance range. These models parameterize, with symmetry constraints, the atomic positions within a unit cell and the atomic displacement parameters (ADPs) that smear these positions due to both thermal motion (temporal variance) and any disorder between local regions (spatial variance). For describing this narrow-*r* range, we assume a simple model where the Ir substructure linearly transitions between the dimerized and un-dimerized configurations (Supplementary Note 3). Using this model, we find that the resolution and signal-to-noise ratio are not high enough to distinguish between Ir dimers being considerably weakened or entirely removed (Supplementary Note 3). This model, implicitly assuming that all the strong dimers in the pumped volume are suppressed by the pump, suggests a pumped phase fraction of ~20%. The small shift between the W signatures of the pumped and equilibrium transition (Fig. 3a) could indicate an inflated Ir ADP due to pumping (Supplementary Note 3) or simply represent a small (~3%) inaccuracy in the intensity normalization of the PDFs.

**Fig. 4:**
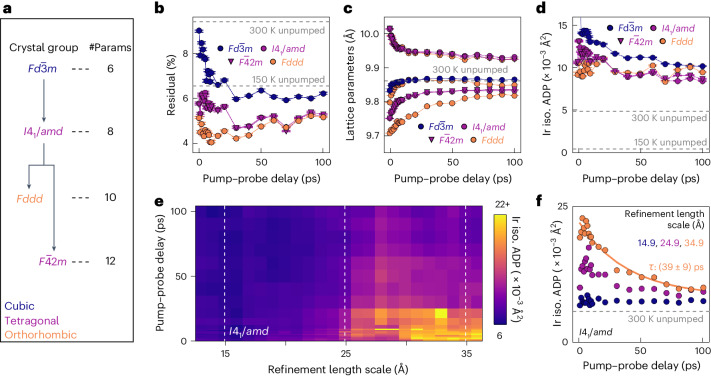
Models of non-local structure. **a**, Crystal space groups used to model the average (8.8–40.0 Å) length scale of the pumped PDFs. The arrows denote a group–subgroup relation. Each subgroup exposes more refinable structural parameters (denoted as #Params). Each model fit also includes four parameters capturing the properties of the experimental setup. **b**, Residual *rw* versus pump–probe delay. The dashed lines indicate reference *rw* for unpumped models at 300 K (${{Fd}}\bar{3}{{m}}$) and 150 K (${{P}}\bar{1}$). **c**, Lattice parameters versus delay. The dashed line indicates the reference lattice parameter for the 300 K equilibrium measurement. **d**, Ir isotropic ADPs versus delay. The dashed lines indicate reference ADPs for unpumped 300 and 150 K measurements. Supplementary Figs. 5 and 6 show an example fit. **e**, *I*4_1_/*amd* isotropic Ir ADPs refined over a sliding window (width, 8.2 Å) to generate a spatial–temporal disorder map. The refinement length scale indicates the centre of the refinement window. **f**, ADPs with a delay for windows at three representative length scales. Longer-length-scale ADPs decrease over time with a time constant of ~40 ps, whereas shorter-length-scale ADPs remain constant. The delay times are marked by the dashed lines in **e**. All the subfigure error bars indicate the uncertainty propagation of photon shot noise in the scattering patterns through to the PDF models.

The small-box methodology is also applied to non-local length scales (8.8–40.0 Å). In this case, the larger-*r* range allows meaningful three-dimensional structural information to be extracted. The experimental PDFs are modelled as a sum of a calculated pumped phase and an assumed static unpumped phase that is defined here as proportional to the measurement at –20 ps pump–probe delay. Including experimental data as a model component will artificially reduce fit residuals *rw* (Methods provides the definition) as this component will, by definition, capture any systematic errors/artefacts present in the data. Accordingly, more focus must be applied to relative differences between pump–probe delays as well as those between different structural models. In the high-temperature equilibrium phase, weak fluctuating dimers exist locally but are uncorrelated over multiple unit cells to average out to a cubic structure in space group ${{Fd}}\bar{3}{{m}}$ at non-local length scales. Applying the ${{Fd}}\bar{3}{{m}}$ unit cell here leads to a larger *rw* over the first 25 ps delay, indicating the underfitting of short-delay data and a lack of model complexity (Fig. 4b). Therefore, the averaged pumped structure is not ${{Fd}}\bar{3}{{m}}$ but a distinct phase in agreement with a previous reflectivity study^22^. These higher residuals are reduced by using a tetragonal structure in space group *I*4_1_/*amd* (a subgroup of ${{Fd}}\bar{3}{{m}}$). This is the unit cell used to describe the high-temperature local ODL structure. At low delays, residuals can be further improved by reducing the symmetry again to an orthorhombic structure in *Fddd* (a subgroup of *I*4_1_/*amd*). The orthorhombic and tetragonal residuals converge at higher delays. Note that both *I*4_1_/*amd* and *Fddd* descriptions are simple high-symmetry models with Ir-atom positions fixed within the unit cell, and any changes in the Ir–Ir distances are dependent on the lattice parameters. Further increasing the complexity of the model, for example, by using a tetragonal ${{F}}\bar{4}$2*m* unit cell that allows some limited changes to Ir-atom positions independent of the unit-cell parameters, offers no further improvement in *rw* and begins to enter the regime of overfitting. Although higher-resolution data, taken with a future higher-energy XFEL or novel detector geometry, may be able to successfully resolve a lower-symmetry model in the future, these data are, therefore, best described non-locally by an orthorhombic unit cell at lower delay times (the prompt phase) and a tetragonal unit cell at longer delay times (the transient phase). The apparent increase in symmetry with delay at non-local length scales reflects an evolution in the longer-range order between the local strong-dimer-removed regions and is consistent with the model-independent analysis. Note that these models provide pumped phase fractions of 20–25% (Supplementary Fig. 7), consistent with the above analysis of the local PDF.

Modelling provides access to information that is not obvious from the raw data alone. For example, both *I*4_1_/*amd* and *Fddd* unit cells involve a new well-defined exponential timescale of (12 ± 1) ps governing the lattice parameters (Fig. 4c, as interpreted below). With the knowledge to carefully search for it, this timescale can be identified in the raw data where it tracks the subtle shifting of some peak positions in Δ*G*(*r*) (Supplementary Fig. 8). Modelling also provides access to ADPs. For the *I*4_1_/*amd* unit cell, the Ir ADPs decrease over tens of picoseconds (Fig. 4d), indicating a reduction in spatial disorder and/or thermal motion, as expected. By re-refining this model over a narrower sliding window of *r*, ADPs can capture how disorder varies as a function of length scale. The model is re-refined using a sliding window with a width of 8.2 Å—a window size that did not risk overfitting. Although the unit-cell structure does not materially depend on the refinement range (Supplementary Fig. 9), the resulting Ir ADPs show a strong dependence (Fig. 4e). This dependence does not correlate with other model parameters and is therefore not a result of overfitting (Supplementary Fig. 9). As a similar strong dependence is also observed for the more complex tetragonal ${{F}}\bar{4}$2*m* model, this is also not a consequence of model underfitting. Below ~25 Å (that is, 2–3 unit cells), the Ir ADPs are easily attributed to thermal effects (~0.006 Å^2^) and show no substantial delay dependence, indicating insignificant laser heating. At larger length scales, the ADPs initially increase by over 350% (to ~0.022 Å^2^) before decreasing with delay with a characteristic time of (39 ± 9) ps (Fig. 4f). This tracks the ~40 ps timescale of the PDF r.m.s. metric (Fig. 2f). At short delays (prompt phase), the pumping of regions separated above a critical distance (seemingly 2–3 unit cells) can be considered independent and their spatial arrangement of bond lengths uncorrelated. This inflates ADPs forced to capture this disorder. The resulting strain between local regions drives lattice relaxation and a (39 ± 9) ps fall in the disorder-related ADP component as the transient phase is approached.

## Discussion

The evolution of non-local (8.8–40.0 Å) pumped CIS from the prompt to the transient structure contains distinct ~12 and ~40 ps timescales governing the lattice parameters and Ir ADPs, respectively. The existence of two seemingly independent timescales can be tentatively explained. The average unit cell averages over the local structure at every point in the pumped material. On initial pumping, there will probably be different local arrangements of atoms that give near-equivalent distributions of nearest-neighbour bond lengths. Our data are not sensitive to these different configurations. These different local structures average to the non-local unit cell, with the proportions of each configuration changing over the ~12 ps timescale. The proportions of local structural configurations give little information on how spatially ordered they are with respect to one another, which is instead tracked by the ~40 ps timescale.

In summary, this uf-PDF investigation has shown that optical pumping suppresses strong Ir–Ir dimers in low-temperature CIS. Despite transitioning between phases with long- and intermediate-range orders, respectively, this optically driven transition is fundamentally characterized by a period of high structural disorder. Above a critical distance (approximately two unit cells), the sub-picosecond suppression of Ir dimers is uncoordinated with local regions taking on different spatial arrangements of bond lengths. We propose that internal strain drives a recovery of order over ~40 ps and an evolution in the average crystallographic structure. This timescale matches the recovery of reflectivity in previous studies^22,23^. The key power of PDF to explicitly isolate ranges of length scales for structural modelling here has allowed disorder, measured through ADPs, to be mapped over both length scales and timescales. This disorder mapping could be widely applied to other non-equilibrium systems.

In agreement with reflectivity studies^22^, we find that pumped CIS is not driven back to the room-temperature equilibrium phase. Instead, the non-local structure at longer delay times is best described using the *I*4_1_/*amd* crystal space group that also describes the local ODL structure at room temperature. It is possible that this phase is related to the ODL state with the fluctuating weak dimers now ordered over long length scales. In similar spinel structures, such as LiRh_2_O_4_ (ref. ^32^), the metal–insulator transition first involves the ordering of fluctuating dimers and then spin dimerization at two distinct temperatures. In CIS, these processes are simultaneous. Speculatively, the non-equilibrium pumped phase may be this otherwise-inaccessible (hidden) intermediate orbitally ordered state lacking spin-singlet dimerization. This phase is distinct from the disordered dimer state achieved by ultraviolet, electron and X-ray irradiation, which locally preserves the dimers^24–27^. We caveat these conclusions by noting that the data, although currently the best available, are limited by a higher minimum *Q*, lower maximum *Q* and broader *Q* resolution than desirable. In a future experiment, it might be possible to distinguish between the symmetries of structural models more robustly and describe the pumped structure with a lower-symmetry unit cell. These current resolution limitations prevent an in-depth structural analysis of the local (sub-unit-cell) structure beyond the key observation that strong Ir dimers are optically suppressed.

Methodologically, this work has successfully demonstrated uf-PDF as a practical and powerful technique given a favourable sample with structural changes pronounced enough to be resolvable at current XFEL facilities. Despite the reduced PDF resolution and shot-to-shot consistency of an XFEL measurement compared with a synchrotron, these are not enough to obscure critical length-scale-dependent structural information. Proven feasible, uf-PDF could be applied to better understand systems, such as VO_2_ (refs. ^14,33^) and 1T-TaS_2_ (ref. ^34^), which also display transient disorder.

This technique will only improve as experimental and data-handling protocols are optimized and higher XFEL energies, possibly in the pipeline for the next decade, increase spatial resolution capabilities. As the picosecond time resolution of this experiment was set by precision expectations for the laser pump delay line, there is no reason why this now-demonstrated technique could not be pushed far into the femtosecond regime. As a complement to studies of diffuse scattering that remain in reciprocal space^14^, uf-PDF is highly quantitative through straightforward comparison with structural models.

## Methods

### Sample synthesis

CIS was prepared by a solid-state reaction in evacuated quartz ampoules. Stoichiometric quantities of metals and elemental sulfur were thoroughly mixed, pelletized and sealed under a vacuum. The ampoules were slowly heated to 650–750 °C and soaked for several weeks with intermediate regrinding and pelletizing. The reaction was deemed complete when no further changes in X-ray powder diffraction scans were observed. The product was found to be a single spinel phase.

### Measurements

A 2–3 μm layer of powdered CIS (grain size, ~1 μm), spread onto Kapton tape, was measured using a transmission pump–probe geometry at the MFX beamline of the LCLS XFEL source. Full Debye–Scherrer rings of the scattered X-rays were collected using a Rayonix MX340 charge-coupled device positioned ~70 mm downstream of the sample (that is, a rapid-acquisition PDF setup^35^) at an acquisition rate of 30 shots per second. An 800 nm, ~100 fs laser pulse from a Coherent Vitara instrument was used to pump the sample with 41 μJ of energy over a 400 μm full-width at half-maximum Gaussian spot. A 23.1 keV X-ray beam probed the sample in ~100 fs pulses. The probed area, a 300 μm top-hat spot, was smaller than the pumped region to minimize any effects from the spatial distribution of pump energy. The sample was cooled using a N_2_ cryostream at a nozzle temperature of 150 K. Each delay time was stroboscopically measured and averaged over hundreds of pump–probe cycles. The pump–probe delays were measured in a pseudo-random order to ensure no accruing permanent structural changes (that is, damage) from repeated pumping. Background measurements of both bare Kapton tape and air scatter were acquired, and dark measurements were taken without the application of the X-ray probe pulse.

### PDF generation

The two-dimensional diffraction image associated with each pump–probe delay time was the average of hundreds of individual stroboscopic measurements. The number of averaged images was not constant with delay due to the varying number of ‘bad shots’ filtered out (where the measurement fails due to a mechanical or electrical fault). Background measurements of air scatter and bare Kapton tape were removed. The images were normalized for the polarization dependence of the detector’s detection efficiency and for any variation in detection efficiency across the detector (the ‘flat field’). An unexpected intensity was noted that was broadband in *Q* and did not vary in intensity around the Debye–Scherrer rings (as expected from the polarization dependence). This was probably the result of multiple scattering events due to the sample thickness. As this did not vary in intensity around the rings like the single-scatter data, it could be removed post hoc. The two-dimensional images were converted into one-dimensional diffraction patterns using the pyFAI software^36^.

Reference unpumped measurements from the Advanced Photon Source synchrotron with higher maximum momentum transfer magnitude *Q* ≈ 23 Å^−1^ were used to guide and confirm detector–sample position calibrations. Typically, diffraction patterns *I*(*Q*) are converted to reduced structure factors *F*(*Q*) and PDFs using the software PDFgetX3 (ref. ^37^). This conversion requires material- and measurement-dependent corrections *F*(*Q*) = *a*(*Q*)*I*(*Q*) + *b*(*Q*) for unknown broadband functions *a* and *b*. PDFgetX3 estimates these functions in an ad hoc manner partially based on the asymptotic behaviour of *F*(*Q*) at high and low *Q* values. Due to the limited measured *Q* range, these estimates were found to be incorrect for the XFEL data by comparison with the unpumped PDFs generated from the synchrotron reference (accounting for different detector properties). Functions *a* and *b* were instead found by comparison with the reference unpumped measurements and fixed for all the delay times. A comparison of synchrotron and XFEL PDFs is shown in Supplementary Fig. 10.

To normalize the diffraction patterns by the average probe X-ray fluence, it is assumed that the total scattering intensity measured is constant with the pump–probe delay. This accounts for both X-ray probe intensity and any change in sample volume due to the possibility of some sample ablation. This self-normalization procedure was verified by comparing the lowest physical PDF peak (Cu–S/Ir–S; Fig. 1d) with the pump–probe delay, which did not meaningfully evolve (as expected; Supplementary Fig. 11).

### PDF fitting

PDF structure fitting was carried out using the diffpy.cmi Python package wrapping the PDFfit2 engine^38^. Structural models were optimized to minimize the root square difference between the experimental *G*_exp_(*r*) and numerical *G*_calc_(*r*) PDFs, as quantified by residual ${rw}\left( \% \right)=100\times \sqrt{\int {[{G}_{\exp }\left(r\right)-{G}_{{{\rm{calc}}}}\left(r\right)]}^{2}{{\rm{d}}r}/\int {[{G}_{\exp }\left(r\right)]}^{2}{{\rm{d}}r}}$. Local and average PDF length scales are separated at 8.8 Å as this threshold does not cut through any PDF peaks. The unpumped reference PDF was defined as the PDF measured with –20 ps pump–probe delay, which could be scaled in magnitude during fitting to account for the fraction of unpumped material. For fits of the average structure, no parameter constraints were applied beyond those required by the crystal symmetry. The presented ADPs represent variance in atomic positions, typically denoted as *U*_iso_, and not the alternative *B* value given by 8π^2^*U*_iso_ also presented in the literature.

## Online content

Any methods, additional references, Nature Portfolio reporting summaries, source data, extended data, supplementary information, acknowledgements, peer review information; details of author contributions and competing interests; and statements of data and code availability are available at 10.1038/s41563-024-01927-8.

## Supplementary information

Supplementary Information Supplementary Figs. 1–11 and Notes 1–3.

## Data Availability

The data presented within this manuscript is openly available at 10.5281/zenodo.11149484 and can be parsed without any specialist software^39^.
