# Author Correction: Resolving length-scale-dependent transient disorder through an ultrafast phase transition

**DOI:** 10.1038/s41563-024-01974-1

**Published:** 2024-07-15

**Authors:** Jack Griffiths, Ana F. Suzana, Longlong Wu, Samuel D. Marks, Vincent Esposito, Sébastien Boutet, Paul G. Evans, J. F. Mitchell, Mark P. M. Dean, David A. Keen, Ian Robinson, Simon J. L. Billinge, Emil S. Bozin

**Affiliations:** 1 Condensed Matter Physics and Materials Science Division, Brookhaven National Laboratory, Upton, NY USA; 2 Department of Materials Science and Engineering, University of Wisconsin, Madison, WI USA; 3 SLAC National Accelerator Laboratory, Menlo Park, CA USA; 4 Materials Science Division, Argonne National Laboratory, Lemont, IL USA; 5 ISIS Neutron and Muon Source, STFC Rutherford Appleton Laboratory, Didcot, UK; 6 London Centre for Nanotechnology, University College London, London, UK; 7 Department of Applied Physics and Applied Mathematics, Columbia University, New York, NY USA

**Keywords:** Phase transitions and critical phenomena, Structural properties, Characterization and analytical techniques

Correction to: *Nature Materials* 10.1038/s41563-024-01927-8, published online 13 June 2024.

Since the version of the article initially published, the Acknowledgments has been amended to state that grant no. DE-FG02-04ER46147, from the US Department of Energy, Office of Science, was given only to P.G.E. and grant nos. DMR-2309000 and DMR-1720415, from the US NSF through the University of Wisconsin Materials Research Science and Engineering Center, were given only to S.D.M.

