# Supplementary material for: Resolving length-scale-dependent transient disorder through an ultrafast phase transition
Source: Nat Mater. 2024 Jun 13;23(8):1041–7. doi: 10.1038/s41563-024-01927-8 (PMC11294184; doi:10.1038/s41563-024-01927-8)
Supplement: Supplementary file 1 — Supplementary Figs. 1–11 and Notes 1–3. [file 41563_2024_1927_MOESM1_ESM.pdf]

nature materials

Supplementary information https://doi.org/10.1038/s41563-024-01927-8

Resolving length-scale-dependent transient disorder through an ultrafast phase transition

In the format provided by the authors and unedited

nature materials

Supplementary information https://doi.org/10.1038/s41563-024-01927-8

Resolving length-scale-dependent transient disorder through an ultrafast phase transition

In the format provided by the authors and unedited

Contents

Supplementary Figure 1 *300 K Pump Response*
Supplementary Figure 2 *Room Temperature Lattice Heating*
Supplementary Figure 3 *PDF Length Scale Ranges*
Supplementary Figure 4 *Confocal Image of Powder CIS*
Supplementary Note 1 *Calculating Difference PDFs from Dimer Breaking*
  Supplementary Note 1 Figure 1 *Difference PDFs from Big Box Modelling*
Supplementary Note 2 *Estimating Uncertainty on Dimer Suppression Factor*
  Supplementary Note 2 Figure 1 *PDF Normalization Uncertainty*
  Supplementary Note 2 Figure 2 *Dimer Suppression Fraction*
Supplementary Note 3 *Interpolating Dimerized and Un-dimerized Unit Cells*
  Supplementary Note 3 Figure 1 *Structural Interpolation*
  Supplementary Note 3 Figure 2 *Interpolating Lattice Vectors*
  Supplementary Note 3 Figure 3 *Dimer Removal Signature Modelling*
Supplementary Figure 5 *Example Pumped PDF Modelled*
Supplementary Figure 6 *100ps $\Delta G(r)$ v. Fit residual*
Supplementary Figure 7 *Non-Local Structural Models*
Supplementary Figure 8 *$\Delta G(r)$ Peak Shifting*
Supplementary Figure 9 *PDF Sliding Window Refinements*
Supplementary Figure 10 *Synchrotron and XFEL PDFs*
Supplementary Figure 11 *Equilibrium Synchrotron Measurements*

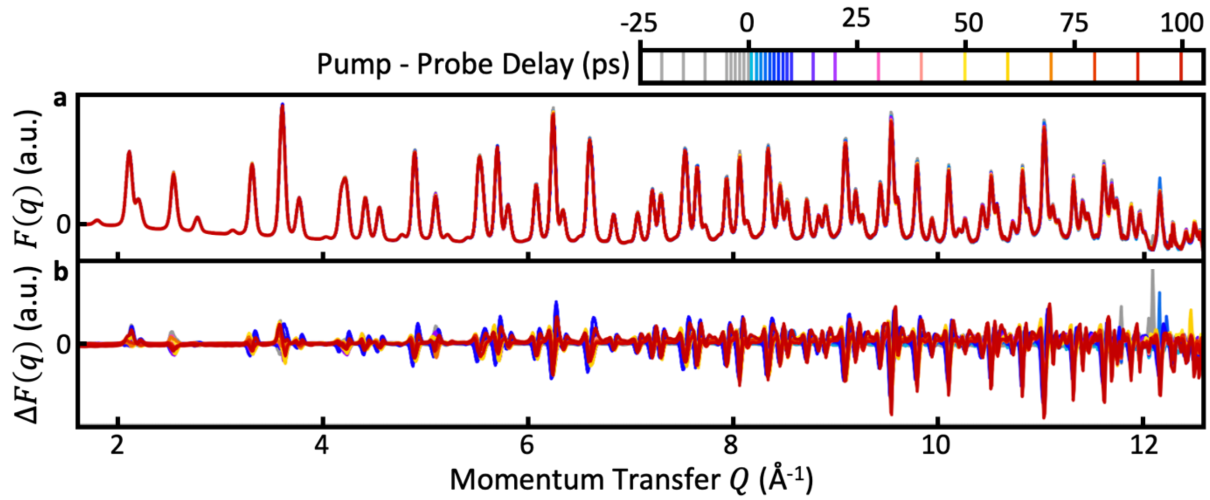

Supplementary Figure 1 | 300 K Pump Response. a) Reduced structure factor $F(Q)$ with varying pump-probe delay at 300 K using 27μJ of pump energy. All negative delay signals (unpumped measurements) are coloured grey. b) $\Delta F(Q)$ subtracting the average of {-20,-15,-10} ps to emphasise differences due to laser pump. Only a mild heating response (seen most clearly by the drop in high Q peak intensities) is seen. Any peak shift due to lattice shifting is much smaller than the peak width.

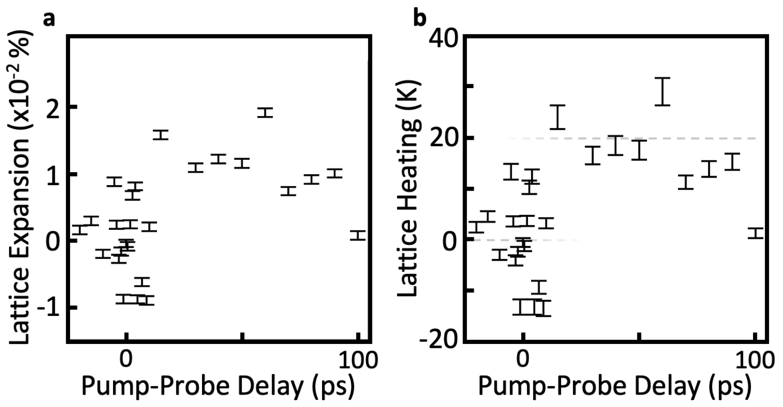

Supplementary Figure 2 | Room Temperature Lattice Heating. a) Thermal lattice expansion of the cubic metallic phase at 300 K under 27μJ of pump laser energy, extracted using the shift of Bragg diffraction peaks. Error bars indicate standard errors derived from maximum likelihood methods, but do not account for systematic effects while converting 2D diffraction images to 1D patterns that may increase uncertainty on these small shifts. b) Using APS synchrotron measurements of the same powder at equilibrium at 250 K and 300 K, this metallic phase is calculated to have a linear thermal expansion coefficient of (6.7 $\pm$ 0.6) x 10^{-6} K^{-1}. Error bars propagate the error from subfigure a with the uncertainty of the thermal expansion coefficient. This converts the lattice expansion into approximately 20 K of laser heating. Note that the heating onset is delayed by approximately 10 ps in accordance with the two-temperature model of lattice heating. During the 150 K pump-probe experiments, 41 μJ of pump energy was used. This would have led to approximately 30 K of lattice heating, leaving the sample still approximately 50 K below the thermal transition temperature.

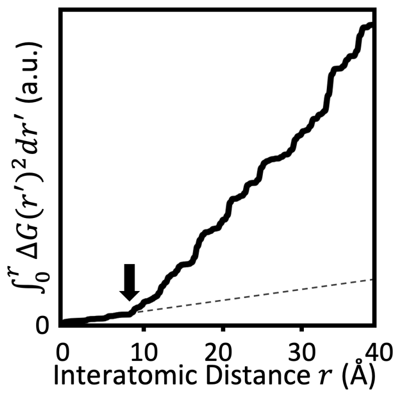

Supplementary Figure 3 | PDF Length Scale Ranges. Cumulative integral of 150 K $\Delta G(r)$ at 100 ps (Fig. 2d). This function is approximately piece-wise linear with an abrupt change in gradient at 9 Å (arrow) encoding a similarly abrupt increase in the magnitude of $\Delta G(r)$ past this distance. Dashed line is a guide to the eye continuing the low r line. If $\Delta G(r)$ was dominated by peak shifting due to lattice heating, this cumulative integral would approximate a smooth parabola rather than two linear segments.

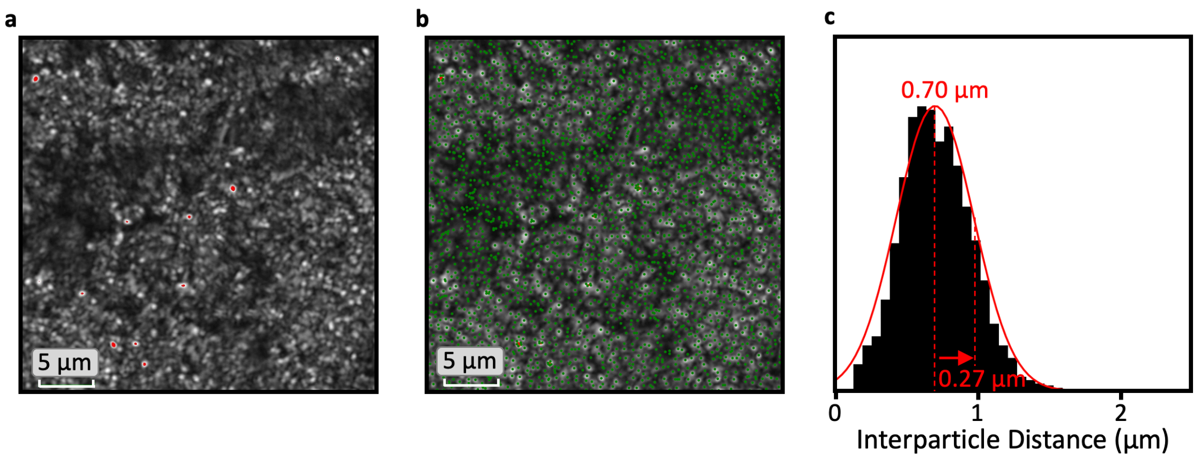

Supplementary Figure 4 | Confocal Image of Powder CIS. a) Confocal microscope image of the powder CIS sample spread in a thin layer on a glass slide. Red points indicate the saturation of the microscope detector. b) The center of each locally bright region, each indicating a powder particle, is marked with a green dot. c) A histogram of distances between each particle and its three nearest neighbors. This is normally distributed with a small shoulder at smaller distances due to minor artefacts in the particle detection (such as around the saturated pixels). In the limit of close packing, this gives particles with a size of (0.7 ± 0.3) µm.

Supplementary Note 1: Calculating Difference PDFs from Dimer Breaking

To calculate the difference PDF $\Delta G(r)$ over a wider r-range from breaking a single Ir-dimer, a large box model of low temperature CIS was formed from a 12 x 12 x 12 supercell of the $P\bar{1}$ unit cell described in the literature[1]. This model contains 27648 Ir atoms. A single pair of dimerized Ir, at an Ir^{4+}[1] and a neighboring Ir^{4+}[4] position (using the literature notation provided in the cited reference) were moved apart by 0.4 Å along the $[1\bar{1}0]$ axis. Each atom was moved an equal distance from their center point. PDFs were calculated for both the unperturbed and altered

structures and from these the difference PDF, $\Delta G(r)$ = $G(r)$ [altered] − $G(r)$ [unperturbed]. This $\Delta G(r)$ consists of a series of delta functions which are positive or negative depending on whether the altered structure has added or removed correlations at a given distance, respectively. These delta functions were then broadened by convoluting $\Delta G(r)$ with a Gaussian function with a width chosen to mimic the resolution of the experimental data. The same procedure was used to calculate $\Delta G(r)$ for a model where the Ir^{4+} ions in *every* dimer were moved apart (both the Ir^{4+}[1] − Ir^{4+}[4] and Ir^{4+}[2] − Ir^{4+}[3] dimers) to completely remove the dimerization within the $P\bar{1}$ structure. The two $\Delta G(r)$ were then scaled such that the intensities of the strong $\Delta G(r)$ peaks below *r* ~ 9 Å were approximately the same as those of the experimental $\Delta G(r)$ at short times. Scale factors of 4320 and 0.55 were used for the single dimer and all dimer breaking models, respectively. The ratio of these scale factors is approximately inversely proportional to the ratio of broken dimers in each model. The difference in correlations between the single dimer model and the unperturbed model become progressively smaller at longer distances; this converts to a calculated $\Delta G(r)$ containing significant noise at high-r because these correlations are multiplied by r when forming $\Delta G(r)$. These two calculated $\Delta G(r)$ are shown in Supplementary Note 1 Figure 1 where they are compared with an averaged experimental $\Delta G(r)$ which is the PDF data taken at 4, 5 and 6 ps minus data recorded at -20, -15 and -10 ps.

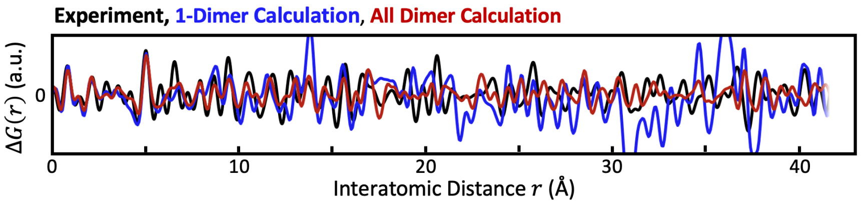

Supplementary Note 1 Figure 1 | Difference PDFs from Big Box Modelling. Calculation of $\Delta G(r)$ from a large supercell of the refined average $P\bar{1}$ structure generated by removing a single Ir-dimer from the structure (blue line) or by removing all Ir-dimers (red line) compared with a representative short-delay time $\Delta G(r)$ (black line). Calculations are scaled by 4320x (blue) and 0.55x (red).

Supplementary Note 2: Estimating Uncertainty on Dimer Suppression Factor

The fraction of Ir dimers (probed by the X-ray pulse) that are suppressed by the pump laser is estimated by comparing the difference PDF dimer removal signature at 3.5 Å between the pumped transition and the equilibrium thermal transition where all dimers are removed. In figure 3, the pumped transition signature used for this purpose averages over all positive pump-probe delays. The reference PDF of dimerized CIS is defined by averaging the unpumped 150 K PDFs at -20, -15 and -10 ps pump-probe delay. Here, uncertainty on the normalization of each XFEL PDF is propagated through to the dimer suppression factor.

The first physical PDF peak (at ~ 2.3 Å, Cu-S / Ir-S) is not expected to change with pump-probe delay. The nearest neighbor distances represented by this peak reflect the strongest bonds in the system which are known to be inert even in case of substantial electron doping (e.g. by substituting Cu with Zn)[2]. However, the height of this peak does vary between PDFs by a few

percent (Supplementary Note 2 Figure 2a,b). These heights can be converted into a surrogate probability density function for the 'mis-scaling' factor on any given PDF by replacing each height value with a Gaussian function of finite width (Supplementary Note 2 Figure 2c). A width of 0.006 is chosen as the smallest width resulting in a function that peaks once only. The function is scaled to give a unity expectation value. Repeatedly generating rescaled versions of the PDFs using samples from this distribution provides measures of uncertainty for both pumped PDFs and the reference dimerized (unpumped) PDF. In Supplementary Note 2 Figure 2a and Figure 3a, each point of the difference PDF is a normal distribution (with unity height) whose width is set by this uncertainty.

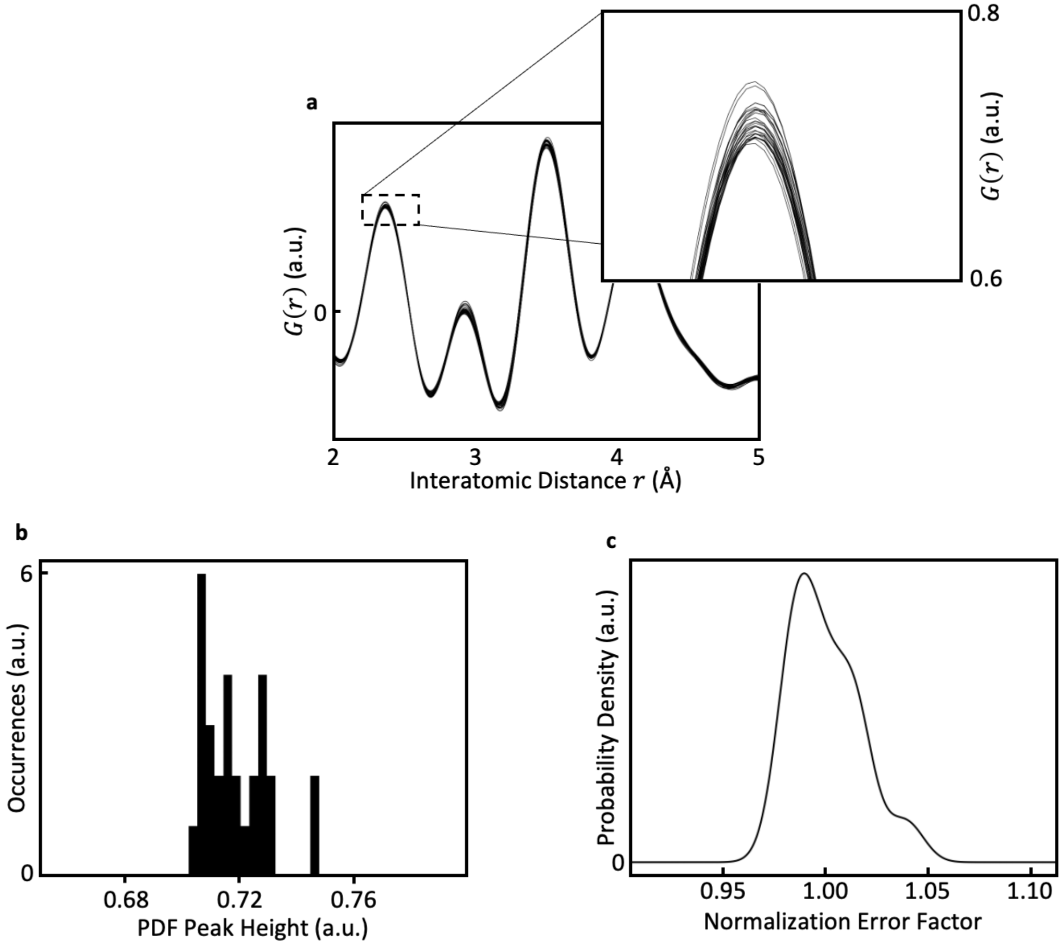

Supplementary Note 2 Figure 1 | PDF Normalization Uncertainty. a) All PDFs overlayed from the pump-probe experiment. The lowest physical peak varies in peak height by a few percent (inset) despite being expected to be constant. b) A histogram of these PDF peak heights. c) A Probability Density Function of the PDF Normalization Error Factor generated by replacing each PDF peak height sample with a Gaussian function of width 0.006. The resulting smooth function is then scaled to a unity expectation value.

If we define the estimated dimer signature fraction to be the difference in scale between the peak of the dimer removal signature between the pumped and equilibrium transitions, we can

use the generated sets of rescaled PDFs to generate a probability density function for this fraction (Supplementary Note 2 Figure 2b, Fig. 3b). This provides a value of $29.4^{+1.0}_{-0.5}$% for the central 68% probability interval.

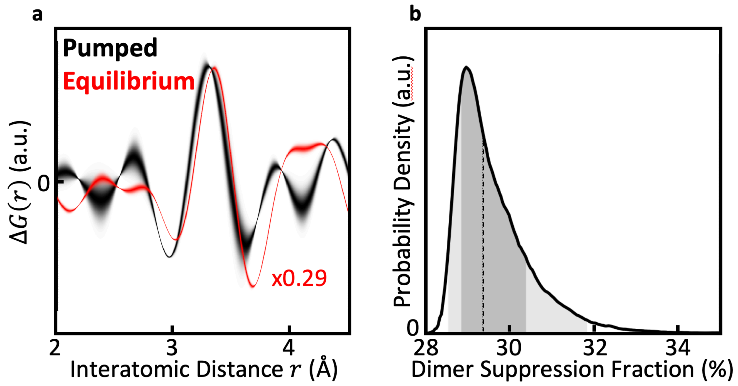

Supplementary Note 2 Figure 2 | Dimer Suppression Fraction. a) Experimental $\Delta G(r)$ for both the pumped (black) and thermally driven equilibrium (red) transitions, showing the dimerized PDF subtracted from the un-dimerized PDF, over the low r range containing the W-signature centered at 3.5 Å of strong dimer removal. The pumped data averages over all positive pump-probe delay times with line thickness indicating uncertainty. The equilibrium data is scaled by x0.29 so that the signatures approximately match in scale. b) Uncertainty on PDF normalization is propagated to a Probability Density Function representing the scaling between the pumped and equilibrium dimer suppression signatures. Dashed line indicated the median value of 29.4%. Shaded areas indicate the central 68% and 95% probability intervals.

Supplementary Note 3: Interpolating between Dimerized and Un-dimerized Unit Cells

We want to construct a simple model of strong dimer removal to compare to the local W signature. The unpumped material is modelled using the known $P\bar{1}$ unit cell that describes equilibrium CIS at low temperature[1]. The structure of equilibrium CIS at low temperature will be denoted here as the *dimerized structure*. The structure of equilibrium CIS at high temperature will be denoted here as the *un-dimerized structure.* To model the pumped material, we interpolate between these dimerized and un-dimerized structures. To be explicit, this means the pumped model has all atoms positioned somewhere between their positions in the dimerized and un-dimerized structures. We can then compare the difference PDF curves between the pumped and unpumped material to the experimental difference curve.

A crystal structure is defined by a set of numerical parameters that will be described here. Interpolating between two structures involves interpolating these parameter values. Importantly, the numerical description of a crystal structure is not unique. This means that meaningfully interpolation first requires the parameterization of the two structures to be chosen to be as numerically similar as possible.

A simple schematic of interpolation between two fictional 2D structures is shown in Supplementary Note 3 Figure 1. The parameters defining a crystal structure include three lattice

vectors defining the unit cell and fractional coordinates (between 0 and 1) for the position of each atom inside the unit cell in the basis of those lattice vectors. Although in general nine variables are required to define three vectors, lattice vectors can be freely rotated (rotating the entire structure) without affecting the calculated PDF or scattering pattern. Therefore, only six variables are needed to describe the lattice vectors: three lattice vector lengths a, b and c and the angles between the lattice vectors α, β and γ. For the P$\bar{1}$ description of the dimerized structure, these are {a: 11.976 Å, b: 7.000 Å, c: 11.956 Å, α: 90.985°, β: 108.513°, γ: 91.033°}. The un-dimerized structure is typically described with a cubic Fd$\bar{3}$m unit cell (a, b, c: 9.831 Å and α, β, γ: 90°) as this is the highest symmetry parameterization. There are many other unit cells, of apparent lower symmetry, that describe the same un-dimerized structure. We search for the unit cell whose parameters are as close as possible to those describing the dimerized structure. This results in a monoclinic unit cell with lattice parameters {a: 12.040 Å, b: 6.951 Å, c: 12.040 Å, α: 90.000°, β: 109.471°, γ: 90.000°}. For this set of lattice parameters, there are many distinct sets of atomic fractional coordinates that still describe the same un-dimerized structure. Again, we pick the set of atomic fractional coordinates that are as close as possible to those in the P$\bar{1}$ description of the dimerized structure.

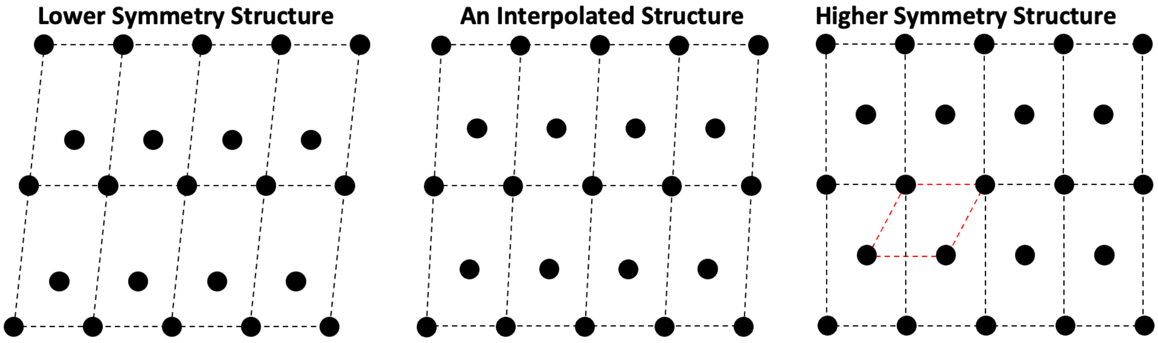

Supplementary Note 3 Figure 1 | Structural Interpolation. Schematic example of interpolation between two fictional 2D structures. The 'Lower Symmetry Structure' has a unit cell containing 2 atoms (black dashed lines). Although the 'Higher Symmetry Structure' has a primitive unit cell containing 1 atom (red dashed lines) it can be described with a very similar unit cell to the Lower Symmetry structure (black dashed lines, containing two atoms) although with orthogonal lattice vectors and the atom in the interior of the unit cell shifted to its centre. The 'Interpolated Structure' mixes these two unit cells, making the lattice vectors of the Lower Symmetry structure more orthogonal and moving the interior atom towards the unit cell centre.

From all possible degenerate parameterizations of the un-dimerized structure, we have selected one such that the numerical values parameterizing it are as similar as possible to those parameterizing the dimerized structure. A new structure can be defined by interpolating these values. For fractional coordinates, this interpolation is trivial. However, trivially interpolating each of the six unit cell parameters can result in unintended modifications to the unit cell volume. Instead, these six parameters must be converted into full lattice vectors

$$\text{Dimerized: }\begin{bmatrix} 11.976 & 0 & 0 \\ -0.1262 & 6.9989 & 0 \\ -3.7964 & -0.2741 & 11.3344 \end{bmatrix} Å, \qquad \text{Un-dimerized: }\begin{bmatrix} 12.04 & 0 & 0 \\ 0 & 6.9513 & 0 \\ -4.0133 & 0 & 11.3514 \end{bmatrix} Å$$

where each row gives a different lattice vector. Arbitrarily, in both cases the first lattice vector is placed along the x-axis and the second vector is place within the x-y plane. Interpolating these 9 values to generate a new intermediate unit cell can still suffer from this volume problem (Supplementary Note 3 Figure 2). By rotating one set of lattice vectors, this problem can be either worsened or improved. We subtly rotate the un-dimerized vectors to remove the volume issue entirely:

$$\text{Dimerized: }\begin{bmatrix} 11.976 & 0 & 0 \\ -0.1262 & 6.9989 & 0 \\ -3.7964 & -0.2741 & 11.3344 \end{bmatrix} Å, \qquad \text{Un-dimerized: }\begin{bmatrix} 12.04 & 0 & -0.012 \\ 0 & 6.9513 & -0.0145 \\ -4.002 & 0.0237 & 11.3554 \end{bmatrix} Å$$

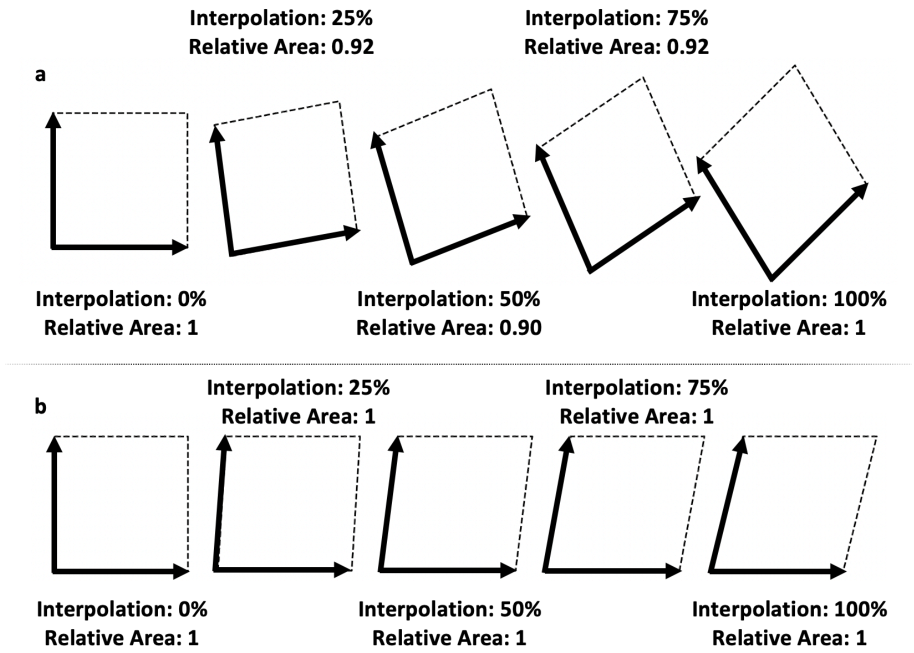

Supplementary Note 3 Figure 2 | Interpolating Lattice Vectors. Two 2D unit cells of equal area are defined by lattice vectors that with a 90° and <90° angles respectively. A) Linearly interpolating the lattice vectors of these two cells can lead to intermediatory unit cells where area is not preserved. B) By rotating the lattice vectors of the two unit cells correctly relative to one another, area is preserved for all possible interpolation values.

We define an interpolated structure intermediate to the dimerized and un-dimerized structures with four interpolation parameters. One controls the interpolation of the lattice vectors while the others describe the interpolation of the fractional coordinates associated with each atomic species (Cu, Ir and S).

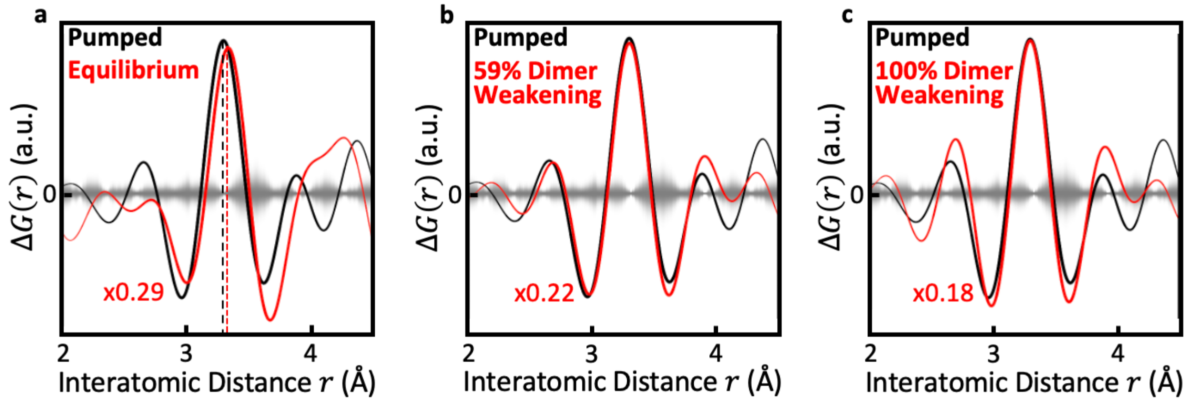

Supplementary Note 3 Figure 3 | Dimer Removal Signature Modelling. a) Experimental $\Delta G(r)$ for both the pumped (black) and thermally driven equilibrium (red) transitions, showing the dimerized PDF subtracted from the un-dimerized PDF, over the low r range containing the W-signature centered at 3.5 Å of strong dimer removal. The equilibrium data is scaled down 3.5x so that the signatures approximately match in scale. The pumped signature peaks at a smaller value of r (dashed lines). Background grayscale gradient indicates the expected noise level, extracted from differences between nominally equivalent negative pump-probe delay measurements and scaled down to account for averaging of the pumped difference. b) Experimental pumped (black) and modelled (red) $\Delta G(r)$. Unpumped modelled PDF given by the known literature structure. Pumped PDF given by interpolating Ir atoms positions towards their un-dimerized configuration to drop the standard deviation of Ir-Ir distances 59%. Ir Atomic Displacement Factor (ADP) inflated by 4.5x for the pumped PDF. Modelled $\Delta G(r)$ scaled down 4.5x to match the experimental data in scale. c) Experimental pumped (black) and modelled (red) $\Delta G(r)$ where Ir atoms in the pumped structure are entirely returned to their un-dimerized configuration and Ir ADP inflated by 6.5x. Modelled $\Delta G(r)$ scaled down 5.5x to match the experimental data in scale.

To best match the average $\Delta G(r)$ for all positive pump-probe delays, the Cu, Ir and S fractional coordinates are interpolated from the dimerized towards the un-dimerized values by 0%, 61.7% and 0.5% respectively. The lattice parameters interpolate 0%. This gives a 59% drop in the spread (standard deviation) of Ir-Ir distances (Supplementary Note 3 Figure 3b). The Ir ADP is inflated 4.5x between the unpumped and pumped PDFs to 0.008 Å^2, shifting the W signature to slightly lower r due to termination effects. Within the noise level, this cannot be reliably distinguished from a model with the Ir fractional coordinates and lattice parameters fixed at 100% interpolation to remove all spread in Ir-Ir distances (Supplementary Note 3 Figure 3c). The Cu and S interpolations optimize to 0 and 7% respectively in this case and the Ir ADP is inflated 6.5x to 0.013 Å^2.

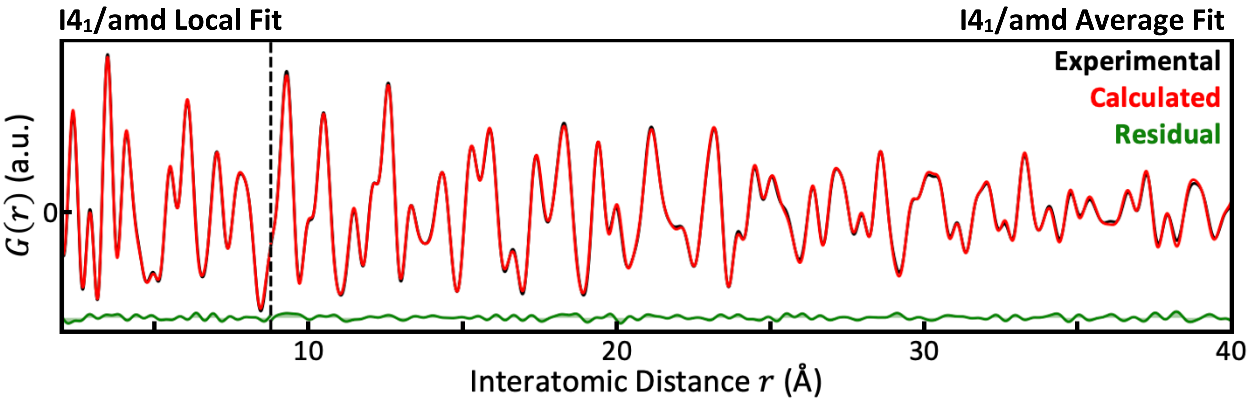

Supplementary Figure 5 | Example Pumped PDF Modelled. Experimental (black) and calculated (red) PDFs for 150 K pumped CIS at 100 ps pump-probe delay using the I4$_1$/amd crystal group. Independent models are applied over the local and average ranges, separated by the vertical dashed line.

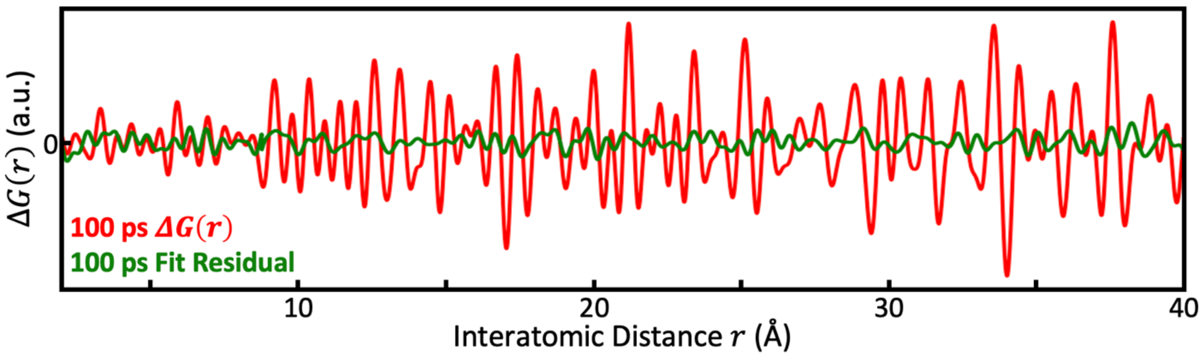

Supplementary Figure 6 | 100ps $\Delta G(r)$ v. Fit residual. $\Delta G(r)$ for the 150 K 100 ps pump probe delay measurement (red) as shown in Figure 2d. For comparison, the fit residual of this PDF is also shown (green) when using the I4$_1$/amd crystal group, as shown in Supplementary Figure 5. The fit residual is significantly smaller in magnitude than the effect of the pump laser, meaning that the model captures well the features of the pumped state.

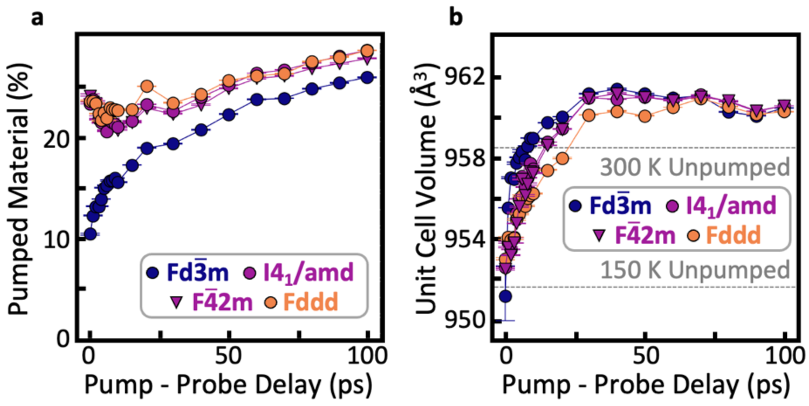

Supplementary Figure 7 | Non-Local Structural Models. Additional model parameters from the small box modelling of the 8.8 – 40 Å. a) The modelled pumped phase fraction with pump-probe delay. This is only significantly different for the Fd$\bar{3}$m unit cell which struggles with underfitting at low delay. b) The unit cell volume with pump-probe delay is consistent for all models. Dashed lines indicate volumes for the 150 K and 300 K unpumped CIS material. All subfigure error bars indicate uncertainty propagation of photon shot noise in the scattering patterns through to the PDF models.

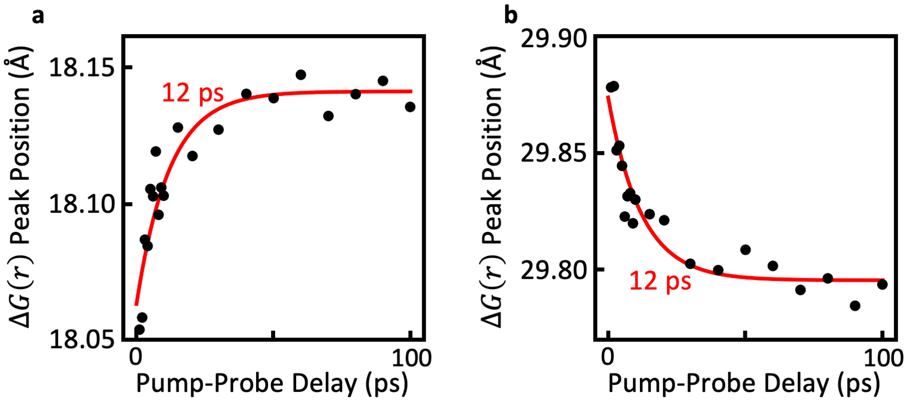

Supplementary Figure 8 | $\Delta G(r)$ Peak Shifting. Some peaks in $\Delta G(r)$ (Fig. 2d) shift subtly with pump-probe delay. This does not indicate simple lattice expansion, as not all peaks shift and those that do can shift to a) higher or b) lower interatomic distances. This shifting matches well to the 12 ps timescale extracted from structural modelling (red lines).

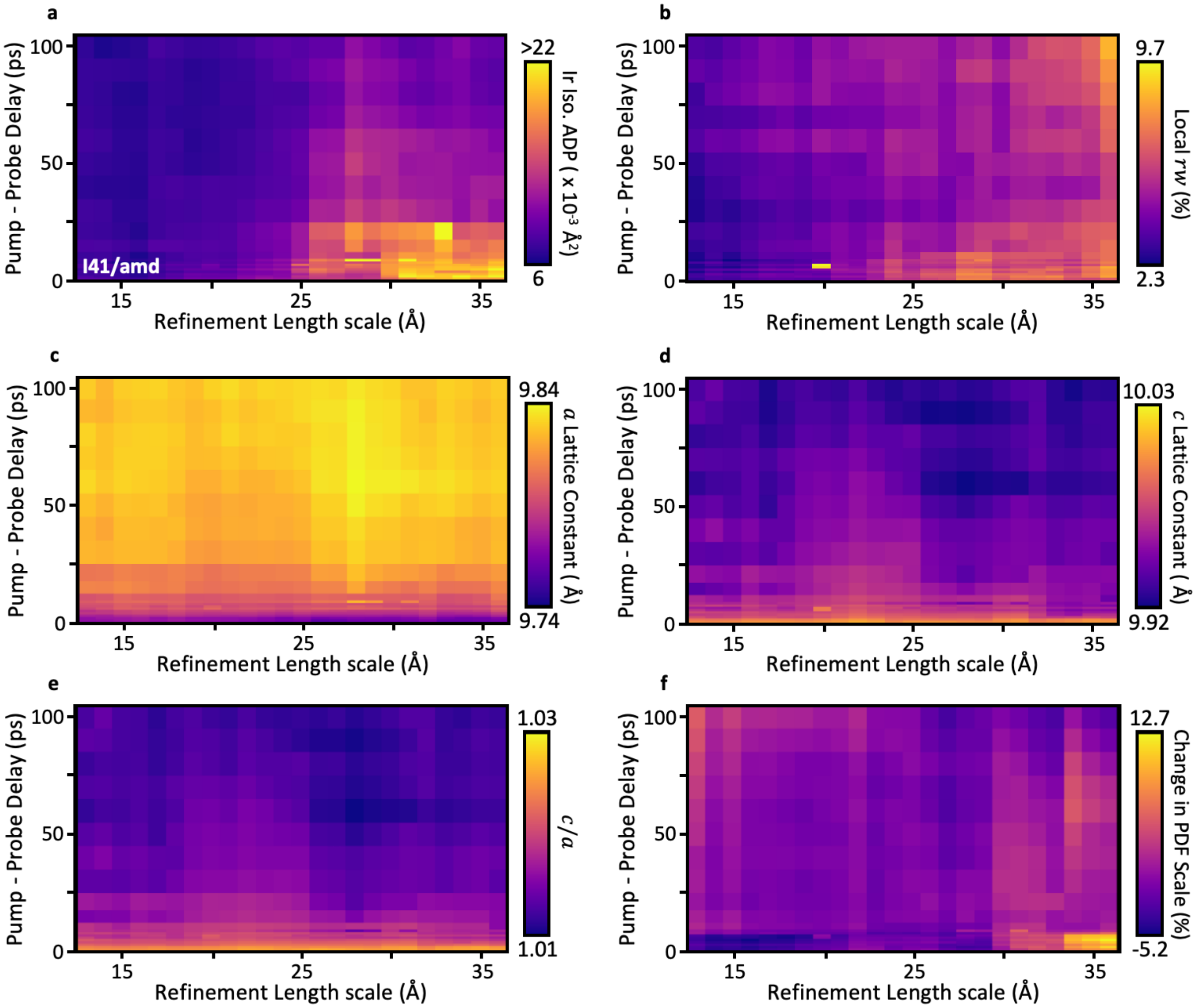

Supplementary Figure 9 | PDF Sliding Window Refinements. $I4_1$/amd model refined over a sliding widow of width 8.2 Å. Refinement Length Scale indicates the center of the refinement window. a) The isotropic Ir ADP as shown in Figure 4e. b) Local residual for each windowed refinement. To ensure this is not an artifact of correlated refinement parameters, the c) a lattice constant, d) c lattice constant and e) a/c ratio do not display changes correlated with the Ir ADPs. f) The scale factor used to match the experimental and modelled PDFs, normalized by the mean average for each pump-probe delay, also does not show the same length scale dependance.

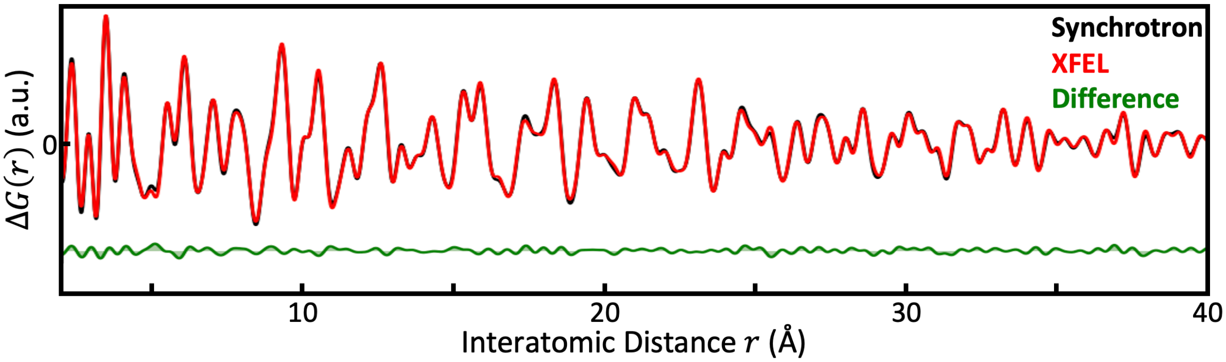

Supplementary Figure 10 | Synchrotron and XFEL PDFs. PDFs of unpumped CIS at 150 K taken at the APS synchrotron (black) and at the LCLS XFEL (red, data is -20 ps pump-probe delay). Green indicates the difference between the two data sets. The synchrotron data is trimmed in reciprocal space to match the momentum transfer range of the XFEL measurement. The XFEL data is corrected with r-dependant scaling term $\propto \exp\left(-0.5 \times (Ar)^2\right.$, where A is a tunable parameter, to account for the different reciprocal space resolutions of the two measurements.

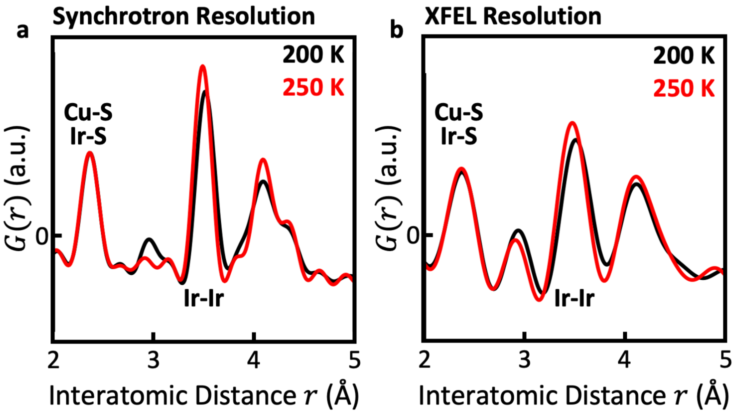

Supplementary Figure 11 | Equilibrium Synchrotron Measurements. a) Unpumped CIS PDF measured at the APS synchrotron above and below the transition temperature using a high maximum Q of 23 $Å^{-1}$. The dimerization signature around the Ir-Ir peak is clear while the first peak (Cu-S and Ir-S) is left insignificantly changed. b) These observations hold when artificially reducing the Q range of this same data to match the XFEL experiment in this work, lowering the PDF resolution.

1. Radaelli, P. G. *et al.* Formation of isomorphic Ir^{3+} and Ir^{4+}octamers and spin dimerization in the spinel $CuIr_2S_4$. *Nature* **416**, 155–158 (2002).
2. Bozin, E. S. *et al.* Local orbital degeneracy lifting as a precursor to an orbital-selective Peierls transition. *Nat Commun* **10**, 3638 (2019).
